# Diaphyseal Aclasis With Pes Anserinus Syndrome

**DOI:** 10.7759/cureus.16548

**Published:** 2021-07-21

**Authors:** Vivek Tiwari, Samir Dwidmuthe, Samrat S Sahoo

**Affiliations:** 1 Orthopaedics, All India Institute of Medical Science (AIIMS), Nagpur, IND

**Keywords:** osteochondroma, pes anserinus syndrome, diaphyseal aclasis, hereditary multiple exostoses, bone tumor

## Abstract

We describe the case of a 20-year-old man who presented with a bony swelling over the medial proximal tibia that caused pain along the pes anserinus tendons, and a history of multiple asymptomatic bony swellings. Wide extraperiosteal resection of the swelling relieved the symptoms with a good outcome within a year. This report describes the pictorial pathoanatomy of a relatively rare association of pes anserinus syndrome caused by osteochondroma in an adult patient. Proximal tibial osteochondromas can also present as pes anserinus syndrome in adult patients with diaphyseal aclasis. Large swellings require wide excision to relieve the stretching pain of pes tendons.

## Introduction

Diaphyseal aclasis, also known as hereditary multiple exostoses or hereditary multiple osteochondromas, is a heritable disorder with autosomal dominant inheritance and presents with multiple bony swellings in long bones as well as flat bones [1]. The clinical findings in these osteochondromas include pain, deformity, limitation of joint movements, or compressive symptoms due to the adjoining vessel/nerve compression [2]. Most such tumors develop around the knee either from the distal femur or proximal tibia [3]. Rarely, proximal tibial osteochondromas can compress the adjoining pes anserinus tendons and cause a constellation of symptoms collectively described as pes anserinus syndrome [4-6]. Though pes anserinus syndrome has been rarely reported with solitary osteochondromas in children, its association with diaphyseal aclasis is even rarer [4]. Moreover, a clear pictorial description of such association in adult patients is sparsely reported in the medical literature. This case report describes pes anserinus syndrome and its pathoanatomical findings caused by proximal tibial osteochondroma in an adult patient with diaphyseal aclasis and whose symptoms were relieved completely after surgical excision with a good outcome after a one-year follow-up.

## Case presentation

**Figure 1:**
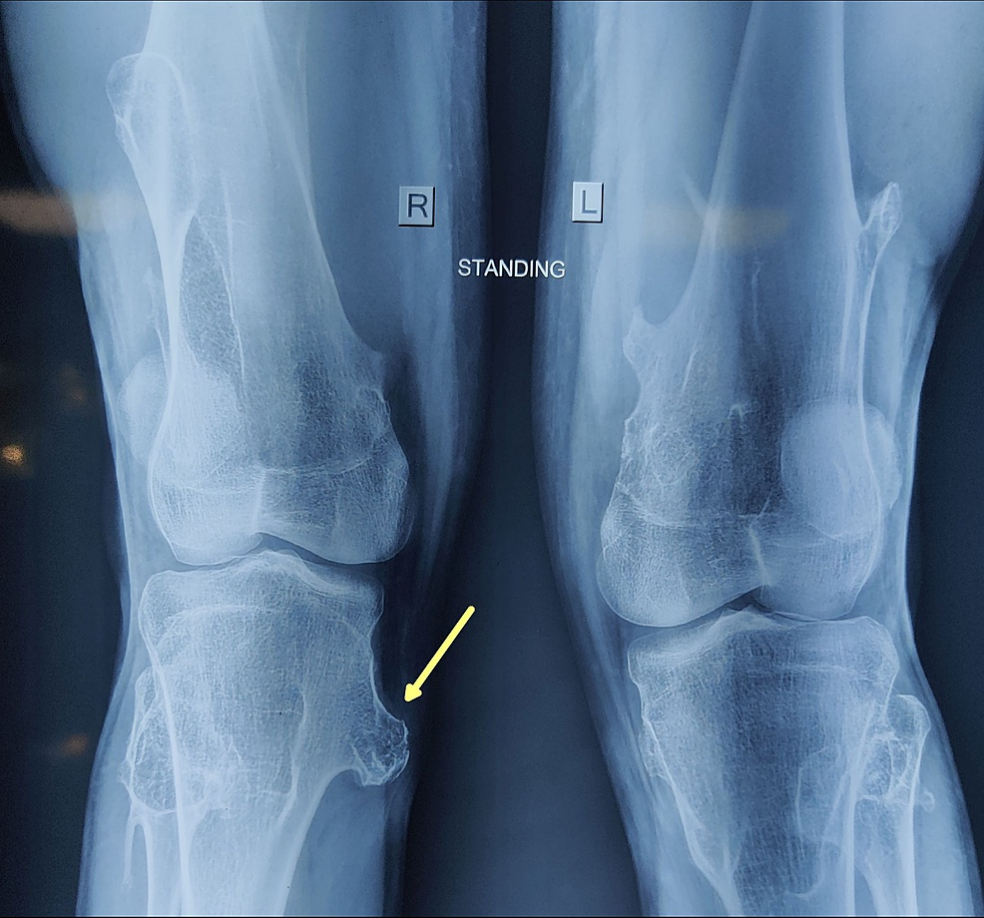
Plain X-ray of both knees anteroposterior view The X-ray shows right medial proximal tibial pedunculated osteochondroma with a stalk (yellow arrow). It also shows small osteochondromas arising from the bilateral distal femur and left proximal tibia.

A 20-year-old man presented with complaints of right upper leg pain that extended over six months, with no relief after taking analgesics. He had a history of multiple asymptomatic swellings in both upper and lower limbs including the forearm, thighs, and the medial aspect of both upper legs since childhood. However, he did not report a rapid increase in any of the swellings. The patient’s father had a similar history of multiple bony swellings since childhood. Examination revealed an immobile, non-tender, 4cm x 2cm swelling fixed to the underlying bone, over the right medial proximal tibia. Also, there was diffuse tenderness along the course of pes anserinus tendons. Plain X-ray demonstrated a pedunculated bony swelling over medial proximal tibia with a tiny stalk without any associated matrix changes/periosteal reaction, pointing away from the knee joint, suggestive of a diagnosis of osteochondroma (Figure 1).

**Figure 2:**
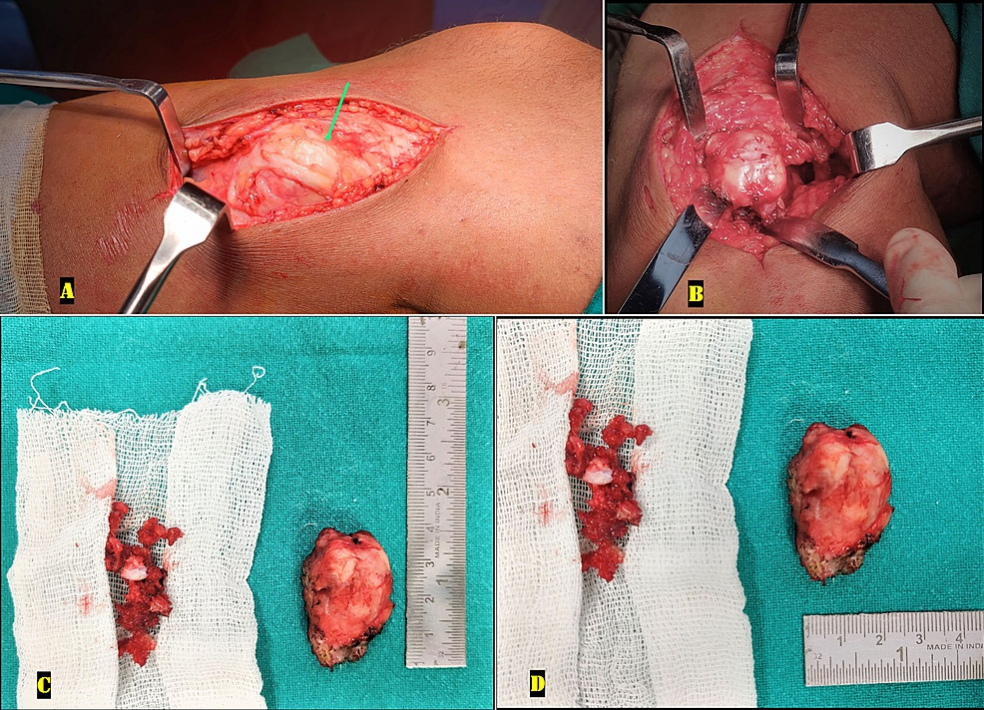
Intraoperative pictures from excision of right medial proximal tibial osteochondroma A: Stretching as well as swelling of the pes anserinus tendons (green arrow) over the bony swelling, B: The osteochondroma with its cartilage cap is exposed after careful dissection of the overlying soft tissues; C: The tumor was excised en masse measuring 4cm in length; the bone nibbling from the tumor bed is also shown, D: The tumor measured 2cm in anteroposterior dimensions (breadth)

Based on the skeletal survey, a presumptive diagnosis of diaphyseal aclasis with pes anserinus syndrome was made, and excision of the painful right proximal tibial swelling was planned. The swelling was excised through a direct medial approach, which revealed swelling of the pes anserinus tendons that were stretched over the bony swelling. The bony swelling was excised extraperiostealy en masse, and the adjoining part of the tibial cortex was nibbled out (Figure 2).

The histopathology report revealed a bony mass with marrow elements in the stalk along with an overlying hyaline cartilage cap without any cellular atypia, confirming the diagnosis of benign osteochondroma. By the end of the year at the last follow-up, the patient was pain-free i.e., measuring zero on the visual analog scale (VAS) with no evidence of recurrence. Consent was obtained from the patient for the publication of this manuscript.

## Discussion

Diaphyseal aclasis is a relatively rare neoplastic disorder with an incidence of around one in 50,000 to 100,000 [7, 8]. It has been associated with a loss-of-function mutation in either exostosin-1 (EXT1) or exostosin -2 (EXT2) genes in >90% of patients [9]. These genes encode glycosyltransferases involved in the synthesis of heparan sulfates. As a result of the mutation, there occurs a deficiency of heparan sulfates that in turn cause various pathoanatomical effects and its clinical presentation in such patients. The spectrum of symptoms in patients with multiple osteochondromas includes limb length discrepancies and limb deformities, spine problems like nerve compression and scoliosis, persistent pains, and various compressive symptoms over adjoining tissues [10]. Impingement of the pes anserinus tendons and impingement over pes anserinus tendons by proximal tibial osteochondromas have been reported in children, and are mostly associated with solitary osteochondromas [4-6]. This report describes the pictorial pathoanatomy of a relatively rare association of pes anserinus syndrome caused by osteochondroma in an adult patient with diaphyseal aclasis.

Semitendinosus, gracilis, and semimembranosus muscles insert over medial proximal tibia in the form of tendinous insertions over the pes anserinus region [11]. Inflammation of the bursa adjoining this region causes pes anserinus syndrome, also known as anserine syndrome or pes anserine bursitis [12]. The typical presentation involves pain along the medial aspect of the upper leg combined with localized tenderness and edema over the pes anserinus insertion. The etiology of this syndrome includes trauma, skeletal abnormalities like genu valgum, infection, foreign body reaction as well as tibial osteochondromas [13]. The diagnosis is mostly clinical in this presentation. However, ultrasonography and MRI have been reported as supporting investigations where doubt in diagnosis remains. The ultrasonographic findings include thickening of the pes anserinus tendons along with fluid in the bursa [14]. MRI has been prescribed in cases with synovial proliferation along with fluid accumulation in the bursa. We neither got ultrasonography nor an MRI as the clinical diagnosis of pes anserinus syndrome could be established with the typical presentation of osteochondroma via X-ray. The treatment extends from activity restriction and limitation, symptomatic management, steroid injection to wide surgical excision in case of large swellings [12].

Fraser et al. described pes anserinus syndrome in 19 children; ten of them were associated with solitary proximal tibial osteochondromas. All cases with osteochondroma required surgical excision. However, none of them had multiple swellings and diaphyseal aclasis [5]. Sakamoto reported pes anserinus syndrome with osteochondromas in five children with two cases of hereditary multiple exostoses. Of the two cases, one was treated with surgical excision with good results. The other had persistent symptoms with conservative management [4]. Tiwari et al. also described pes anserine bursitis in three pediatric cases with proximal tibial osteochondroma. The lesions presented in these cases were resolved with conservative management [6]. In this report, we have pictorially described the actual pathological changes seen in pes anserinus tendons, of their stretching and swelling in an adult patient diagnosed with diaphyseal aclasis who developed pes anserine bursitis secondary to a large pedunculated proximal tibial osteochondroma. The swelling, being large in size, required wide excision to relieve the symptoms and prevent a recurrence.

## Conclusions

This report describes the pictorial pathoanatomy of a relatively rare association of pes anserinus syndrome with osteochondroma in an adult patient. Proximal tibial osteochondromas can also present as pes anserinus syndrome in adult patients with diaphyseal aclasis. Large swellings require wide excision to relieve the stretching pain of pes tendons.
